# On the use of programmable hardware and reduced numerical precision in earth‐system modeling

**DOI:** 10.1002/2015MS000494

**Published:** 2015-09-18

**Authors:** Peter D. Düben, Francis P. Russell, Xinyu Niu, Wayne Luk, T. N. Palmer

**Affiliations:** ^1^ AOPP, Department of PhysicsUniversity of OxfordOxfordUK; ^2^ Department of ComputingImperial College LondonLondonUK

**Keywords:** weather forecast, numerical precision, programmable hardware, FPGA, Lorenz '95, dynamical core

## Abstract

Programmable hardware, in particular Field Programmable Gate Arrays (FPGAs), promises a significant increase in computational performance for simulations in geophysical fluid dynamics compared with CPUs of similar power consumption. FPGAs allow adjusting the representation of floating‐point numbers to specific application needs. We analyze the performance‐precision trade‐off on FPGA hardware for the two‐scale Lorenz '95 model. We scale the size of this toy model to that of a high‐performance computing application in order to make meaningful performance tests. We identify the minimal level of precision at which changes in model results are not significant compared with a maximal precision version of the model and find that this level is very similar for cases where the model is integrated for very short or long intervals. It is therefore a useful approach to investigate model errors due to rounding errors for very short simulations (e.g., 50 time steps) to obtain a range for the level of precision that can be used in expensive long‐term simulations. We also show that an approach to reduce precision with increasing forecast time, when model errors are already accumulated, is very promising. We show that a speed‐up of 1.9 times is possible in comparison to FPGA simulations in single precision if precision is reduced with no strong change in model error. The single‐precision FPGA setup shows a speed‐up of 2.8 times in comparison to our model implementation on two 6‐core CPUs for large model setups.

## Key Points

- Huge saving in computing cost via reduced numerical precision in earth‐system modeling
- Long and short‐term simulations have similar level of minimal numerical precision
- Numerical precision can be reduced with time in a weather forecast simulations

## 1 Introduction

Numerical models in Computational Fluid Dynamics (CFD) that do not resolve the whole range of the turbulent energy cascade can only be approximations of reality since the dynamics of a continuous fluid is reduced to a finite representation on a numerical grid in space and time. Many models in CFD work with double‐precision floating‐point arithmetic since double precision will almost guarantee that rounding errors do not influence the quality of model simulations for many applications. There is no doubt that a strong reduction in numerical precision will reduce the quality of results. However, papers that study the use of so‐called inexact or approximate hardware in geophysical modeling suggest that it would be possible to reduce energy consumption and to increase performance significantly if high numerical precision is sacrificed, with only a small impact on model quality [*Düben et al*., 2014a, 2014bb; *Düben and Palmer*, 2014]. Higher performance and reduced energy consumption with inexact hardware will potentially enable simulations with higher resolution for the same computational budget, therefore, improving results for many applications.

The basic idea to trade precision against computational cost in hardware development has been discussed for several years [*Palem*, 2003, 2005] and several different approaches to inexact hardware have been investigated. The first approach studied so‐called “stochastic processors.” Here power consumption is reduced significantly by reducing the applied voltage to the floating‐point unit. However, hardware errors can occur within numerical simulations if voltage is decreased [*Narayanan et al*., 2010; *Kahng et al*., 2010]. A second approach is “pruning” in which the physical size of the floating‐point unit is reduced, by removing parts that are either hardly used or do not have a strong influence on significant bits in the results of floating‐point operations [*Lingamneni et al*., 2011; *Düben et al*., 2014a, 2014bb]. Pruning of the floating‐point unit can also be combined with the use of inexact memory and cache [*Düben et al*., 2015]. A third approach is to use hardware that allows flexible precision floating‐point arithmetic, such as Field Programmable Gate Arrays (FPGAs). FPGAs are integrated circuit designs that can be customized by the user (“programmable” hardware). While stochastic processors, pruning, and inexact memory and cache are still in an early stage of research and not yet realized in general purpose computing systems, FPGAs could already prove their usefulness in multiple applications. For designs targeting FPGAs, designers use hardware languages such as Verilog and VHDL to capture the customized operations in hardware. The hardware descriptions go through vendor tool chains to be synthesized into configuration bit streams, which are downloaded into the configuration memory of FPGAs to define the implemented hardware. In practice, the ability to construct customized hardware designs on postfabrication chips is often the principal drive behind the adoption of FPGA technology. One of the key factors exploitable by FPGA designs is the ability to trade the accuracy of computational results with silicon area, power, and operating frequency. The number of bits used to represent fixed or floating‐point numbers can be reduced to a minimum (the hardware can be used in an “information efficient” way, see *Palem* [2014]). Development tools that can automatically optimize design accuracy have been proposed [*Lee et al*., 2006; *Boland and Constantinides*, 2010, 2012]. However, the target accuracy is set based on user experience.

The high computational efficiency of FPGAs makes their use interesting for CFD applications. Recent studies reveal that the use of FPGAs shows huge potential for atmospheric modeling. Oriato et al. show a speed‐up of a meteorological limited area model of up to a factor of 74 on a dataflow node (which is based on FPGAs) compared to a X86 CPU computing node [*Oriato et al*., 2012]. Gan et al. run a global shallow water model on four FPGAs with a 330 times speed‐up over a 6‐core CPU [*Gan et al*., 2013]. We note that it is extremely difficult to make fair comparisons between hardware which is as different as CPUs and FPGAs. Performance measures will change if different generations of hardware are considered and performance results are highly problem specific.

In this paper, we investigate the performance of a nonlinear application with relevance for atmospheric modeling on FPGA hardware. The main focus is on testing how performance can be increased if precision is reduced below single precision. The use of FPGAs enables us to study the trade‐off between precision and performance on real hardware. A comparable study is not possible with CPU or GPU devices for high‐performance computing, since they would only support standard single and double‐precision floating‐point arithmetic.

For efficient use of inexact hardware, it is of vital importance to estimate the minimal level of precision that allows model simulations with no significant increase in model error. However, the chaotic nature of geophysical models makes it a difficult challenge to find this level of precision. If the model configuration is changed only slightly, results of simulations will diverge from results of the control simulation over time. Every change in precision—even an increase—will cause a significant change in the prognostic variables if integrated over a long‐enough time period.

To identify the minimal level of precision for models that are computationally cheap, it is always possible to perform a trial‐and‐error approach in which precision is reduced until the model error is increased significantly. One can do this by running several simulations at different precision levels and plotting the mean error for a prognostic quantity against the number of bits used to represent, for example, floating‐point numbers. Ideally, this error is evaluated against the true system (e.g., atmospheric observations), but it can also be calculated against a reference system (e.g., a double precision simulation). However, such an approach will be very expensive for large model configurations, especially if different parts of the model will accept different levels of precision. For complex models that consist of hundreds of thousands of lines of code and run on hundreds or thousands of processors in parallel, such as weather and climate models, this would be prohibitively expensive. In this paper, we identify the level of minimal numerical precision that can be used with no strong increase in model error for different simulation intervals (between 1 and 100,000 time steps). We discuss the need for trial‐and‐error tests with expensive, long‐term simulations.

In the Ensemble Prediction System of the European Centre for Medium‐Range Weather Forecasts (ECMWF), the first 10 days of a forecast are calculated with the highest affordable resolution on the local supercomputer (T639/32 km). After 10 days, the forecast is continued with lower resolution (T319/64 km) to save computing resources [*Directorate*, 2012]. However, the change of resolution and the truncation of the numerical fields induce perturbations and imbalances. If numerical precision is reduced with time and with increasing model error, this might allow a reduction of computational costs for forecast simulations at the end of the forecast period with no introduction of imbalances due to a change in resolution. In this paper, we will test if such an approach is beneficial for a model that represents a chaotic system.

This paper focuses on three scientific problems:

1. We attempt to construct a strategy to estimate the minimal level of precision that can be used in complex numerical models of chaotic systems that avoids expensive trial‐and‐error simulations.
2. We investigate if a reduction of numerical precision with increasing forecast time is a useful method to reduce computing time and power consumption in weather predictions.
3. We test if a reduction in precision is an efficient method to save computing time for simulations of chaotic systems on FPGA hardware in the context of geophysical fluid dynamics.

To approach these problems, we investigate the two‐scale, chaotic Lorenz '95 model on FPGA hardware. The Lorenz model has been used in previous work [*Ott et al*., 2004; *Wilks*, 2005; *Kwasniok*, 2012; *Sapsis and Majda*, 2013; *Arnold et al*., 2013] to test conceptual ideas, for example, for data assimilation or parametrization schemes and to investigate the chaotic and multiscale behavior of atmospheric dynamics. Our main motivation for investigating such a model is that it shows chaotic, multiscale behavior in a high‐dimensional, complex model configuration while effectively being one dimensional. The model resembles many properties of a numerical model in CFD since it is based on grid points that interact in a similar way to finite difference discretization schemes. The main disadvantage of using the Lorenz model is that it is a toy model with no real‐world system to compare results with. A fair comparison that would increase numerical resolution when numerical precision is reduced and compare the model error that is verified against a truth for precise and imprecise simulations at the same computational cost is not possible. Therefore, we need to define an artificial range for an acceptable “model error.” This can be realized by taking simulations with slight perturbations of parameters as a truth. Differences between the perturbed parameter simulations and the standard configuration will serve as an estimate for model error. To this end, we assume that the main parameters of the system are only known down to a precision of 1%. We argue that a parameter uncertainty of 1% is realistic for most numerical simulations in CFD. For comparison, we also perform simulations for which the small‐scale variables of the two‐scale model are parametrized. The difference between the parametrized and the original Lorenz system serves as an approximation of errors of model simulations at strongly reduced resolution.

Sections 2 and 3 provide information on the Lorenz model and the experimental setup. Sections 4–6 approach the three scientific problems described above. Section 7 summarizes results and concludes the paper.

## 2 A Chaotic Toy Model

**Figure 1:**
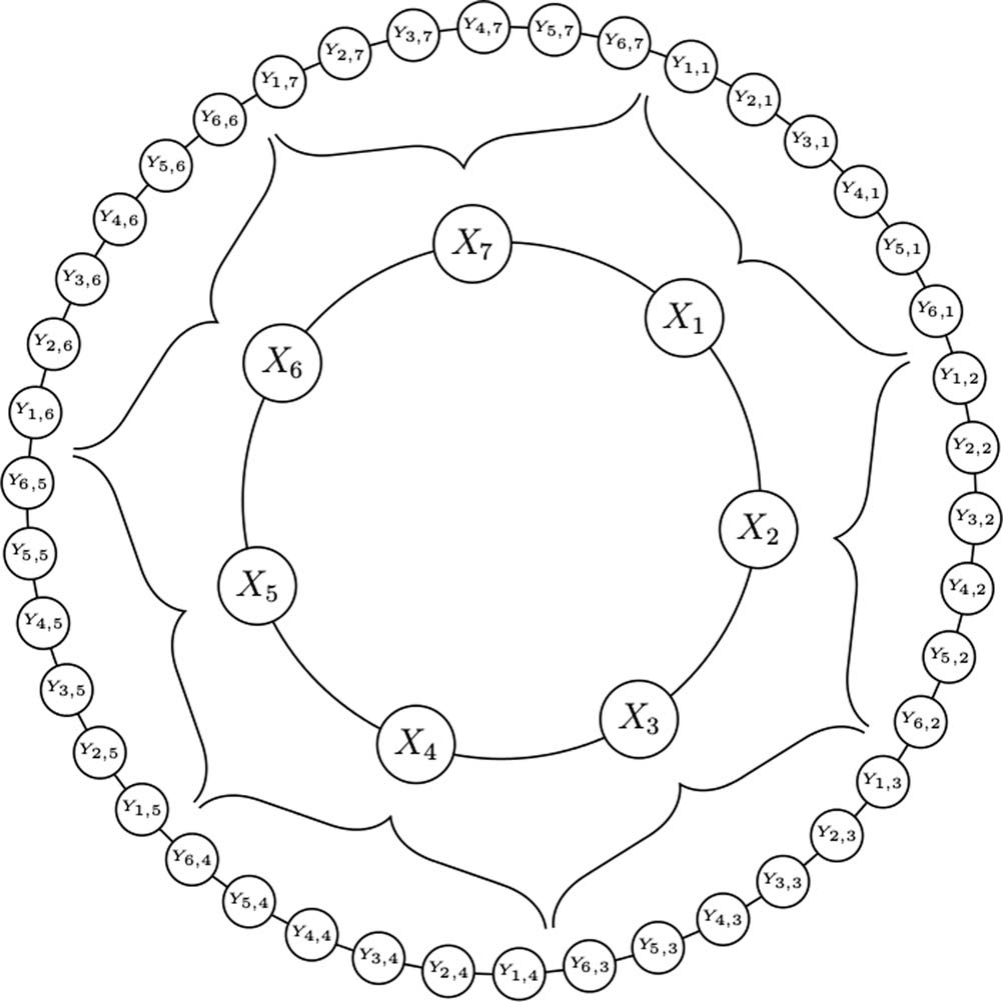
Schematic of the model configuration with *N_x_* = 7 and *N_y_* = 6.

The Lorenz '95 model was proposed in *Lorenz* [2006] and consists of two sets of prognostic variables that form one‐dimensional periodic spaces and interact with each other via coupling terms. The *X* and *Y* variables of the model represent dynamics on large and small scales, respectively. Figure 1 provides a sketch of the model configuration. We use *N_x_* large‐scale variables *X_k_* ( $X_{k-N_{x}}=X_{k}=X_{k+N_{x}}$), and *N_y_* small‐scale variables $Y_{j,k}$ ( $Y_{j,k-N_{x}}=Y_{j,k}=Y_{j,k+N_{x}},\;Y_{j+N_{y},k}=Y_{j,k+1}$ and $Y_{j-N_{y},k}=Y_{j,k-1}$) for each *X_k_*.

The standard configuration of the Lorenz model uses only 36 large‐scale *X* quantities that are each coupled to 10 small‐scale *Y* quantities. Such a configuration is certainly too small for a meaningful performance analysis on an FPGA. However, since there is no restriction for the number of degrees of freedom, we increase the size of the used model heavily.

The model system is described by the following set of equations:

(1) $$\frac{dX_{k}}{dt}=-X_{k-1}\left(X_{k-2}-X_{k+1}\right)-X_{k}-\frac{hc}{b}\sum_{j=1}^{N_{y}}Y_{j,k}+F,$$

(2) $$\frac{dY_{j,k}}{dt}=-cbY_{j+1,k}\left(Y_{j+2,k}-Y_{j-1,k}\right)-cY_{j,k}+\frac{hc}{b}X_{k},$$

where *F*, *h*, *b*, and *c* are constants. Time will be measured in Model Time Units (MTUs).

We use two different configurations for simulations. The first configuration with $N_{x}=20,000$, *N_Y_* = 128, *F* = 30, *h* = 1, *b* = 10, and *c* = 4 is denoted **C4**. The second configuration with $N_{x}=20,000$, *N_Y_* = 64, *F* = 40, *h* = 1, *b* = 10, and *c* = 10 is denoted **C10**. The use of either *c* = 4 or *c* = 10 will cause a smaller or larger time scale separation. We use an explicit Runge‐Kutta time stepping scheme of fourth order and a time step of Δt=0.0005 MTUs.

A chaotic model can be evaluated in two different time frames that represent predictability of the first and the second kind: if the specific state of the system is of interest (for example, in weather forecasts), simulations need to be initialized a short time before the actual event since predictability will decrease quickly with increasing time due to model errors and uncertainties in initial conditions. If a mean state of the system is important (for example, in climate simulations), initial conditions do not matter after a long spin‐up and model results will need to be averaged over a long time period. We investigate both types of simulations for the Lorenz model and study short‐term simulations that are integrated for up to 4 MTUs (8000 time steps) and long‐term simulations of 100 MTUs (100,000 time steps for the model run + 100,000 time steps for the spin‐up). We evaluate the mean forecast error for the large or small‐scale quantities compared to the control simulation for short‐term simulations and the probability density functions of the large‐scale quantities for long‐term simulations. All short‐term simulations are initialized from initial conditions that are provided by a long‐term control simulation in single precision.

To obtain an estimate for a realistic error range, we compare simulations of the standard configuration with reduced precision with simulations with perturbed parameters that have *c* and *F* changed by 1%. The difference between the perturbed and unperturbed simulations is compared against the change due to reduced precision. For comparison, we also show results with parametrized model configurations that remove the small‐scale quantities from the model and replace the missing scale interactions in the equation for the large‐scale quantities with a parametrization term (see Appendix A for a detailed derivation). The model configuration with parametrized small scales is obviously much cheaper compared to the full model configuration due to the strong reduction in the number of degrees of freedom.

## 3 Experimental Setup and Details

The achieved performance of an FPGA design is dependent on a number of factors including the clock rate at which a design can be compiled and the extent to which design parallelism can be increased before running out of resources (digital signal processing elements (DSPs), memory, or on‐chip logic) or becoming impossible to route. Floating‐point calculations can be implemented either by digital signal processing elements on‐chip or via chip logic. Furthermore, high utilization of DSPs in the design does not necessarily indicate higher computational throughput, as this can also occur due to space/latency trade‐offs in the design. Peak performance is therefore difficult to establish. For the FPGA hardware used in this work, 2016 DSPs exist on chip each of which is capable of performing one FLOP per clock cycle ideally. Typical clock rates range from 150 to 300 Mhz. If arithmetic intensity or on‐chip resources are not a limiting factor, it is possible for an FPGA design to process data at the rate of the underlying memory system.

In this paper, the FPGA hardware designs were captured using Maxeler's MaxJ language and compiled with MaxCompiler version 2013.2.2. They were compiled to run on a MAX3A Vectis Dataflow Engine containing a Xilinx Virtex 6 SXT475 FPGA. The MAX3A was hosted in a Maxeler MPC‐C500 computing node and connected via PCI express. All designs were compiled to run at 150 MHz. The design permits global and local‐scale quantities to have independent bit widths for significand and exponents, subject to some restrictions imposed by the Maxeler tools.

Software simulations are run on the machine containing MAX3A cards and two 6‐core Intel Xeon X5650 processors running at 2.67 GHz, giving 12 cores in total. Each socket has a 12 Mbit level‐three cache. The machine has 48 Gbit of memory and is running Centos Linux 6.3. While the results of the performance evaluation in section 6 will be specific to the used Maxeler hardware, the results of the other sections will be the same (or at least very similar) for other reconfigurable systems which support flexible floating‐point precision.

The floating‐point representations on the FPGA resemble those defined by the IEEE 754 standard used by most modern processors with the primary difference being no support for denormalized numbers. IEEE 754 values possess a sign bit and a fixed number of bits assigned to represent significand and exponent values. For example, a double‐precision value has 52 bits assigned to the significand, 11 to the exponent, and 1 to the sign bit. Due to floating‐point normalization, the significand bits are considered to have a leading “1” bit that is not stored. Hence, we consider double precision to have a 53 bit significand.

Düben et al. showed that precision can be reduced more in the calculation of the small‐scale quantities compared to the precision of the large‐scale quantities to obtain the same quality of results in atmospheric modeling [*Düben et al*., 2014a, 2014bb; *Düben and Palmer*, 2014]. Therefore, we will reduce precision for the large (equation (1)) and the small‐scale dynamics (equation (2)) independently. We denote the number of bits in the exponent of floating‐point numbers that are used to calculate all floating‐point operations in equations (1) and (2) for the large and small‐scale quantities with **EX** and **EY**, respectively. The number of bits used to represent the floating‐point significand for the equations of the large and small‐scale quantities are denoted **PX** and **PY**. Although precision can be customized to application needs on the FPGA, not all combinations of **EX** and **PX** are possible on the used hardware. For **EX** = 4, **PX** needs to be ≤5, for **EX** = 5, **PX** needs to be ≤13 (the same constraints hold for **EY** and **PY**). All control simulations are calculated in single precision. Differences between single and double precision simulations are negligible for all diagnostics considered.

## 4 Simulations With Reduced Precision

In this section, we perform model simulations with reduced numbers of bits in the significand and exponent. Precision will be reduced for the representation of large or small‐scale dynamics (**PX**, **EX** or **PY**, **EY**, respectively), or both. Section 4.1 provides results for long‐term simulations with reduced numerical precision. Section 4.2 presents similar tests for short‐term simulations. In section 4.3, we try to find the minimal level of precision that can be used for combinations of **EX**, **EY**, **PX**, and **PY** with no strong increase in model error. Section 4.4 will calculate the model error for single precision and reduced precision model simulations if a simulation with perturbed parameters is taken as “truth.”

### 4.1 Long‐Term Simulations With Reduced Precision

**Figure 2:**
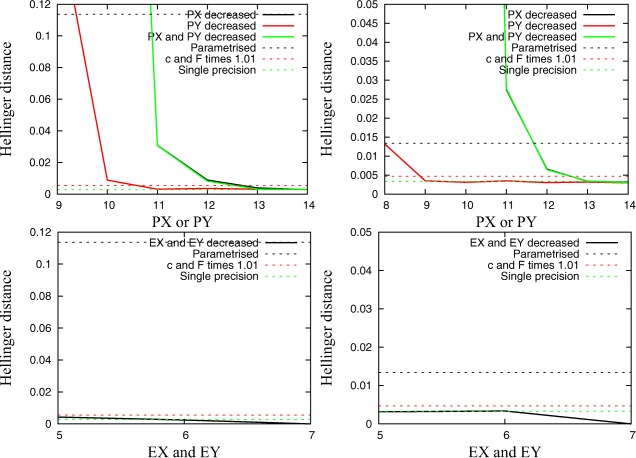
Hellinger distance of large‐scale quantities between a control simulation in single precision and simulations with reduced precision plotted against (top) the number of bits in the significand (**PX** and **PY**) and the (bottom) number of bits in the exponent (**EX** and **EY**) for (left) the **C4** and (right) the **C10** configuration. The horizontal lines in the plot mark Hellinger distances for simulations with perturbed parameters or parametrized small scales as well as a single precision simulation with different initial conditions that represents the measurement error. Note that the number of bits in the significand (**PX** and **PY**) is reduced to 13 bits for the run with **EX** = **EY** = 5 since **EX** = **EY** = 5 and **PX** = **PY** > 13 is not possible on the given hardware.

Figure 2 shows the Hellinger distance between the control simulation in single precision and simulations with reduced precision. The Hellinger distance is a measure of the difference between two probability density functions (PDFs). PDFs describe the mean state of the system. If the Hellinger distance is calculated between two single precision simulations that use different initial conditions, the result can be used as a measure for the measurement error at the given level of statistics.

A reduction of **PX** to only 12 bits (11 bits for **C4**) will lead to a smaller change in the probability density function of the large‐scale quantities than parametrizing the small‐scales quantities. A reduction of **PX** to only 13 bits will cause a smaller change in the probability density function compared to perturbed parameters. If **PY** is reduced, the change of the probability density function for the large‐scale quantities is smaller compared to the impact of a reduction in **PX**. A reduction of **PY** to 10 bits (less than 8 bits for **C10**) will cause a smaller change than parametrizing small‐scale quantities. A reduction of **PY** to 11 bits (9 bits for **C10**) creates an error that is smaller compared to the influence of a parameter perturbation. A simultaneous reduction of **PX** and **PY** causes an error that is almost identical to the error with only **PX** reduced.

A simulation with an exponent reduced to 5 bits (**EX** and **EY**) shows a smaller error compared to a simulation with perturbed parameters. A further reduction of the number of bits in the exponent is not feasible since this would not allow a representation of the significand with more than 5 bits on the given hardware. For **EX** = **EY** = 7, the Hellinger distance is exactly zero for two simulations with the same initial conditions. This indicates that simulations are bit reproducible and that the additional range of exponents is not used.

### 4.2 Short‐Term Simulations With Reduced Precision

**Figure 3:**
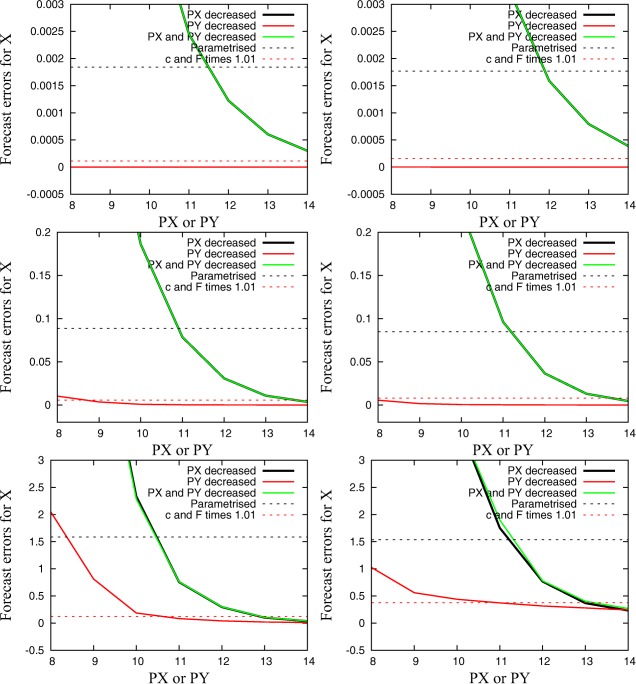
Mean forecast error for large‐scale quantities plotted against the used precision level for **PX** and/or **PY** at different times (after 0.0005, 0.025, and 0.5 MTUs from top to bottom) with (left) the **C4** and (right) the **C10** configuration.

Figure 3 shows the mean forecast error for large‐scale quantities plotted against the precision used for **PX** and/or **PY**. After only one time step (0.0005 MTUs), results appear to be more sensitive to **PX** compared to the long‐term simulations in the previous section. However, after 50 time steps (0.025 MTUs), results are already comparable to results from long‐term simulations and show that simulations with **PX** reduced to 12 bits (11 bits for **C4**) still obtain a lower forecast error than parametrized simulations. Simulations with **PX** reduced to 14 bits show smaller errors than parameter perturbations. A change of **PY** has no immediate influence on the forecast error of the large‐scale quantities. After one time step, the error is almost zero. After 0.025 MTUs (50 time steps), the error of the simulation with **PY** = 9 is smaller than the error due to parameter perturbations. However, the error exceeds the error for parameter perturbations for **PY** smaller than 11 after 0.5 MTUs (1000 time steps).

**Figure 4:**
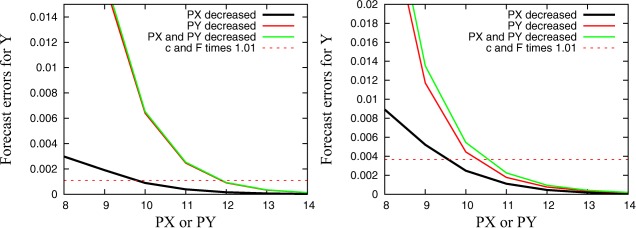
Mean forecast error for small‐scale quantities plotted against the used precision level for **PX** and/or **PY** after 0.025 MTUs with (left) the **C4** and the (right) **C10** configuration.

Figure 4 shows plots similar to those in Figure 3 for the forecast error of the small‐scale quantities. Results are fairly different since the forecast error is initially much larger for a reduction of **PY** compared to a reduction of **PX** due to the slow response via interaction terms. The forecast error is comparable to the change due to parameter perturbations if precision for **PY** is reduced to 12 bits (11 bits for **C10**). We found a strong sensitivity to reduced precision after one time step and that the influence of a reduced **PX** becomes more important with increasing lead time (not shown here), analogous to the results in Figure 3.

We conclude that forecasts of only very short time periods provide valuable information on the precision level that is acceptable in expensive long‐term simulations. However, two features of short‐term forecasts need to be recognized: (1) simulations of only a couple of time steps are more sensitive to reduced precision than longer simulations, probably because perturbations of rounding errors will have a mean close to zero if averaged over a large number of time steps; (2) interactions between small and large‐scale quantities need a certain amount of time to propagate the perturbations of the small‐scale quantities to the dynamics of the large‐scale quantities (approximately 50 time steps = 0.025 MTUs for the Lorenz model). However, if the forecast error is evaluated for each quantity individually, results from simulations for only 50 time steps could provide useful information on the minimal level of precision that can be used with no significant increase in model error. Results after 50 time steps do not deviate by more than 2 bits compared to results from longer forecasts (e.g., 2 MTUs) or long‐term simulations (e.g., 50 MTUs). These very short simulations could be performed in an automatized trial‐and‐error way in which precision is reduced until the minimal acceptable level of accuracy is reached.

### 4.3 Benchmark Tests With Reduced Precision

In this section, we present results for simulations that try to reduce precision for combinations of **EX**, **PX**, **EY,** and **PY** as far as possible with no significant impact on model results. Tests in section 4.1 revealed that **EX** and **EY** can be as small as 5 with no strong change in forecast quality. **PX** and **PY** can be as small as 14 and 12 bits, respectively, to cause smaller errors than simulations with a parameter perturbation and as small as 13 and 10 to cause a smaller error than simulations with parametrized small scales. Therefore, we will perform simulations with **EX** = 6, **PX** = 14, **EY** = 5, and **PY** = 12 and **EX** = 5, **PX** = 13, **EY** = 5, and **PY** = 10.

**Figure 5:**
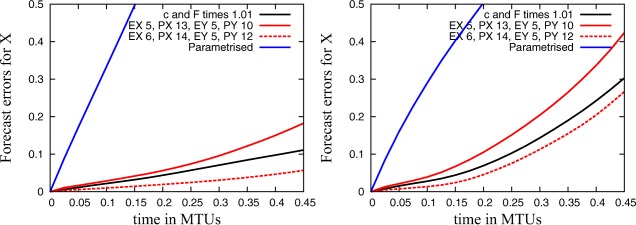
Mean forecast error for large‐scale quantities between the control simulation in single precision and (1) simulations with reduced precision, (2) simulations in single precision with perturbed parameters, and (3) simulations with parametrized small‐scale quantities with (left) the **C4** and (right) the **C10** configuration and plotted against time.

Figure 5 and Table 1 present results for short‐term forecast errors and the Hellinger distance of large‐scale quantities for simulations with reduced precision and simulations with single precision that have either perturbed parameters or parametrized small‐scale quantities compared to the control simulation in single precision. The simulations with reduced precision to **EX** = 5, **PX** = 13, **EY** = 5, and **PY** = 10 show a Hellinger distance and a forecast error which is a bit higher than the error with perturbed parameters. The forecast error with **EX** = 6, **PX** = 14, **EY** = 5, and **PY** = 12 is smaller than the error with perturbed parameters and the Hellinger distance is at the same level as the measurement error. In the performed simulations, all results with reduced precision are much better compared to simulations with parametrized small scales.

**Table 1:** Hellinger Distance for Simulations With Reduced Precision, Perturbed Parameters, and Parametrized Small‐Scale Quantities in Comparison to Standard Single Precision Simulations for the **C4** and the **C10** Configuration^a^

Run	Hellinger Distance **C4**	Hellinger Distance **C10**
Single precision, changed init. cond.	0.0028	0.0033
c and F times 1.01	0.0054	0.0047
**EX** = 5, **PX** = 13, **EY** = 5, **PY** = 10	0.0079	0.0033
**EX** = 6, **PX** = 14, **EY** = 5, **PY** = 12	0.0029	0.0030
Parametrized	0.1137	0.0134

a The table also presents an estimate for the measurement uncertainty via the Hellinger distance for a single precision simulation with changed initial conditions.

### 4.4 Model Error With Inexact Hardware

**Figure 6:**
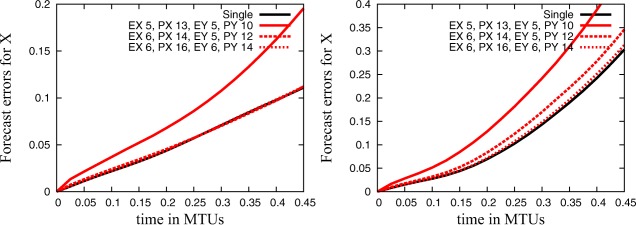
Mean forecast errors for large‐scale quantities for simulations with reduced and single precision with the standard parameters for (left) configuration **C4** and (right) configuration **C10** plotted against time. Simulations with parameter perturbation (*c* and *F* times 1.01) are taken as truth.

In previous subsections (sections 4.1–4.3), all diagnostics evaluated the change of model dynamics due to reduced precision in comparison to simulations in single precision. In this section, we will evaluate the influence of reduced precision on model error in comparison to a “truth” to obtain a more realistic setup for model forecasts. There is no real‐world reference system for the model under investigation. However, we can assume that the perturbed parameter configuration represents the true system and present the Hellinger distance and the forecast error for different precision levels calculated against this truth in Figure 6 and Table 2. While the forecast error is clearly increased if we use a precision level that has been found to produce a larger error than the difference between the truth and the model configuration (see section 4.3 for **EX** = 5, **PX** = 13, **EY** = 5, **PY** = 10), the increase in the forecast error is only very small if we use a precision level that has been found to produce a lower error (see **EX** = 6, **PX** = 14, **EY** = 5, **PY** = 12). If we increase precision by 2 bits for each significand (see **EX** = 6, **PX** = 16, **EY** = 6, **PY** = 14), the reduced precision hardly influences the forecast error for both configurations. The Hellinger distances agree well for all simulations except for **EX** = 5, **PX** = 13, **EY** = 5, **PY** = 10, and **C4**. Here the Hellinger distance is increased but still in a reasonable range (less than 50% increase) when compared to the Hellinger distance of the simulation with perturbed parameters.

**Table 2:** Hellinger Distance Between Simulations With Reduced Precision or Single Precision and Simulations With Perturbed Parameters for the **C4** and the **C10** Configuration

c and F Times 1.01	Hellinger Distance **C4**	Hellinger Distance **C10**
Single precision	0.0054	0.0047
**EX** = 5, **PX** = 13, **EY** = 5, **PY** = 10	0.0115	0.0045
**EX** = 6, **PX** = 14, **EY** = 5, **PY** = 12	0.0049	0.0041
**EX** = 6, **PX** = 16, **EY** = 6, **PY** = 14	0.0063	0.0043

## 5 Precision Reduced With Time

In this section, we investigate if it is feasible to reduce numerical precision with time in forecast simulations. We reproduce the situation of a weather forecast that compares an imperfect model against the true system (the atmosphere) by taking the configurations with perturbed parameters in single precision as “truth.” The standard model represents an imperfect model for which model forecasts diverge from the truth with time. We will perform several sets of simulations with the imperfect model that reduce precision at different time steps to either **EX** = **EY** = 6 and **PX** = **PY** = 12 or **EX** = **EY** = 5 and **PX** = **PY** = 13. We know from the results in section 4 that both configurations will increase forecast errors.

**Figure 7:**
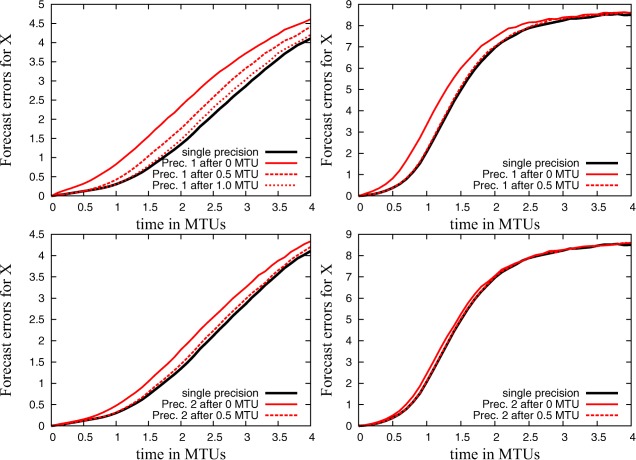
Short‐term forecast error for simulations with single precision and simulations for which precision is reduced to either (top) **EX** = **EY** = 6 and **PX** = **PY** = 12 (Prec. 1) or (bottom) **EX** = **EY** = 5 and **PX** = **PY** = 13 (Prec. 2) after 0, 0.5, or 1.0 MTUs for the configuration (left) **C4** and (right) **C10**.

Figure 7 shows the results. If precision is reduced from the beginning of the simulations, the forecast error is clearly increased compared to simulations in single precision. However, if the level of precision is reduced only after 0.5 MTUs (1.0 MTUs for simulations with configuration **C4** and **EX** = **EY** = 6 and **PX** = **PY** = 12), the forecast error is much smaller and of a similar magnitude as in the single precision simulation. An approach that reduces precision with increasing lead time in a weather forecast appears to be promising.

**Figure 8:**
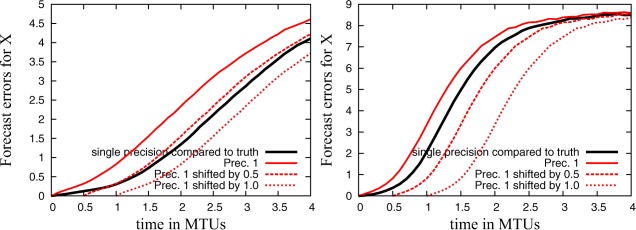
Short‐term forecast error for simulations with an imperfect model compared against the truth and forecast error for simulations of the imperfect model and reduced precision compared against simulations of the imperfect model in single precision. For the reduced precision simulations, the same graph is plotted three times, shifted in the *x* direction by 0, 0.5, and 1.0 MTU for the configuration (left) **C4** and (right) **C10**.

Figure 8 compares the forecast error between the imperfect model and the truth against the forecast error due to reduced precision. While the error of the single precision simulation with the imperfect model is calculated against the truth, the error of the imperfect model with reduced precision is compared against the imperfect model simulation in single precision. The error due to reduced precision is larger than the model formulation error if perturbations start from the beginning of the simulation (continuous red line). If, however, precision is only reduced after 0.5 or 1.0 MTUs, this is effectively a shift of the error contribution of reduced precision to the right (dashed and dotted red line). If the error due to reduced precision (with or without time shift) exceeds the model formulation error—see the plots that are not shifted in time and the plot for **C4** shifted by 0.5 MTUs—model forecasts will almost certainly be influenced if the imperfect model is integrated with reduced precision since it is unlikely that interactions between the model formulation error and rounding errors will improve model simulations. For all other plots, the model formulation error in single precision exceeds the error due to reduced precision. To get no degradation of results, it appears to be a necessary condition to make sure that the shifted plots of the reduced precision simulations are lower compared to the plot of the model error in comparison to the truth in Figure 8. A comparison to Figure 7 reveals that this necessary condition, that will only need one model simulation with reduced precision, performs well for the given setup.

## 6 Performance Evaluation

This section investigates possible speed‐ups when precision is reduced. See *Russell et al*. [2015] for a more detailed evaluation of the performance increase and energy savings of the used model configurations. If precision is reduced on the FPGA, the simulation can be rearranged such that more small‐scale degrees of freedom can be calculated in parallel on the given size of the FPGA. While eight *Y* variables can be read per clock cycle in single precision (denoted by **8Y** in the following), we can read 16 *Y* variables at the precision **EX** = 6, **PX** = 16, **EY** = 6, **PY** = 14 (**16Y**) and 24 at the precision **EX** = 5, **PX** = 13, **EY** = 5, **PY** = 10 (**24Y**). Table 3 shows the speed‐up results for two different sizes of the model system with $N_{x}=1600$ and $N_{x}=819,200$. A simulation with $N_{x}=819,200$ has more than 100 million degrees of freedom and provides realistic speed‐up results for applications of the size of a full weather or climate model. Overheads due to I/O and PCI‐express data transfer are subtracted and not considered in timings.

**Table 3:** Relative Speed‐Up for Simulations on the FPGA and With CPUs^a^

Relative Speed‐Up	$N_{x}=1600$	$N_{x}=819,200$
*CPU*		
Double precision, 12 cores	0.45	0.18
Single precision, 1 core	0.04	0.03
Single precision, 6 cores	0.24	0.18
Single precision, 12 cores	0.47	0.36
*FPGA*		
Single precision, **8Y**	1.00	1.00
**EX** = 6, **PX** = 16, **EY** = 6, **PY** = 14, **16Y**	1.90	1.91
**EX** = 5, **PX** = 13, **EY** = 5, **PY** = 10, **24Y**	2.46	2.46

a Simulations are either performed with $N_{x}=1600$ or with $N_{x}=819,200$. We use *N_y_* = 144 for both configurations.

The FPGA configurations scale extremely well with the number of degrees of freedom. To give one example, the single precision setup updates 1.14E+09 degrees of freedom per second for both the $N_{x}=1600$ and the $N_{x}=819,200$ setup. The performance is increased by a factor of 1.91 or 2.46 in comparison to the single precision simulation if precision is reduced. We know from benchmark tests in section 4.4 that the configuration with **EX** = 6, **PX** = 16, **EY** = 6, **PY** = 14 shows hardly any increase in model error while the configuration **EX** = 5, **PX** = 13, **EY** = 5, **PY** = 10 shows a small increase in model error.

The table also provides results for simulations of the model on CPU hardware. The CPU simulations were executed on a dual‐socket machine with each socket containing a 6‐core Intel Xeon X5670 processor (12 cores in total) running at 2.67 GHz with Hyperthreading enabled. The implementation was written in C++ and compiled with version 12.1.4 of the Intel C++ compiler. OpenMP was used for intranode parallelization and the hwloc library was used to pin threads to physical cores. A “first‐touch” policy was used for memory initialization such that memory was allocated in the appropriate NUMA domain for each thread. Fundamental differences between CPU and FPGA architectures make it difficult to generalize results beyond the specific problem being studied. Our CPU implementation adopts a standard domain‐decomposition strategy for parallelization in which each thread computes a subset of results with partitioning occurring in the *K* dimension. Inter‐thread communication is independent of problem size, depending only on the stencil size of the derivative computation. Threads synchronize after each substep of the Runge‐Kutta update. Each Runge‐Kutta increment is calculated in sequence and accumulated into the output state.

We note that the classic Runge‐Kutta update is problematic for performance of CPUs due to memory bandwidth requirements. For a Runge‐Kutta update, our working set size (the amount of storage used for an update) is 3 times our system state size and consists of storage for the input state, the updated state and a temporary used for storage of Runge‐Kutta increments. The amount of memory read and written for an update are 9 times and 7 times the size of the system state, respectively. We believe this to be consistent with a typical implementation of classic Runge‐Kutta time stepping.

Using the STREAM benchmark, we rate the maximum practical achievable bandwidth of our test system at around 35,000 MiB/s. Our CPU implementation achieves 4.61E+08 degree of freedom processes per second, where each is represented by a single‐precision value. Considering the 16 reads/writes of each degree of freedom performed, our implementation achieves approximately 80% of this.

In contrast, in the FPGA implementation, reads and writes of the input, temporary, and output states become links in a pipeline between kernels implementing each of the Runge‐Kutta steps. Hence, the FPGA only reads and writes the system state to and from memory once—the theoretical minimum.

Much work exists on Runge‐Kutta schemes that attempt to reduce the amount of intermediate storage required which is important on systems where memory is limited [*Ketcheson*, 2010]. However, on modern memory hierarchies, reducing memory bandwidth utilization is of high importance for achieving maximum performance. Fusion of the Runge‐Kutta increment calculations into a single traversal of the system state would theoretically enable a reduction of the amount of data written to and from main memory. Such a transformation is called *loop fusion* by compiler developers [*Bacon et al*., 1994].

The Lorenz '95 implementation resembles finite difference codes in the manner that derivative calculations involve stencil‐like operations. This makes fusion of the Runge‐Kutta steps difficult to correctly achieve by hand, requiring code duplication and complex iterations patterns in order to respect dependencies. Such code could be implemented using a code generator or using C++ template metaprogramming. We therefore do not claim that our Lorenz '96 implementation achieves the peak performance that is possible on the architecture we have chosen, but that it is consistent with the performance of a reasonably optimized implementation.

On the CPU implementation of the model, single precision simulations are approximately a factor of 2 faster compared to double precision simulations for large model configurations. For simulations of large model setups ( $N_{x}=819,200$), single precision simulations on the FPGA are a factor of 1.0/0.36≈2.8 faster compared to single precision simulations on two 6‐core CPUs. Russell et al. showed that a single precision simulation with the same Lorenz '95 model setup on an FPGA was 10.4 times more power efficient compared to a CPU simulation with 12 cores in parallel. If precision is reduced, such that 16 or 24 “Y” variables can be read per clock cycle (as for the two reduced precision configurations considered), power efficiency is increased to a factor of 18.9 and 23.9, respectively [*Russell et al*., 2015]. However, all comments about difficulties in the comparison between CPU and FPGA hardware that apply for the performance comparisons above also apply for the comparison of energy consumption.

## 7 Conclusions

We analyze a two‐scale, chaotic model on FPGA hardware. On the used hardware, trade‐offs between precision and performance are investigated. The used model exhibits important properties of large‐scale simulations of weather and climate since it shows nonlinear/chaotic behavior and scale interactions. We scale the size of the model to the size of a high‐performance application to provide meaningful performance results for full global circulation models of atmosphere or ocean using up to 100 million degrees of freedom in our simulations. We show that precision can be reduced substantially in our model simulations before rounding errors cause a significant increase in model error. Results confirm conclusions from previous publications: a reduction in precision for large‐scale dynamics has a stronger impact on model quality than a reduction of precision when calculating small‐scale dynamics. The following three paragraphs summarize the results on the three scientific problems that are listed in section 1 and therefore the results of sections 4–6, respectively.

First, we investigate how a reduction in precision will influence the quality of short and long‐term simulations and how to relate errors due to reduced precision with model errors. We conclude that the minimal level of precision that should be used is similar in short and long‐term simulations and argue that simulations of only a small number of time steps (e.g., 50, this is equivalent to a couple of hours for an atmosphere model) can provide valuable information about the minimal level of accuracy that can be used in both weather (e.g., 1000 time steps) and climate‐type forecasts (e.g., 100,000 time steps). This result is consistent with the approach of seamless predictions in atmospheric modeling for which short‐term simulations over days, weeks, or seasons (initial value problems) are used to understand and improve long‐term climate simulations (boundary‐value problems, see, for example, *Palmer et al*. [2008]). While trial‐and‐error tests can be avoided for expensive integrations if only short‐term simulations are evaluated in a first approach to reduced precision, we find that it is important to evaluate each reduced‐precision simulation parameter against its own reference value, if the model simulation is evaluated after only a small number of time steps; this is necessary since interactions between different quantities will need an appropriate amount of time to propagate perturbations. Furthermore, simulations with very small numbers of time steps (e.g., 1) are very sensitive to reduced precision, probably since rounding errors will have a mean close to zero if averaged over an appropriate number of time steps. The difference between the double precision and the reduced precision simulation can be represented as a forcing terms added to the equations of motion. To identify the minimal level of precision that can be used with no strong reduction in forecast quality for earth‐system models, we believe that a comparison between the magnitude of this rounding error forcing and the magnitude of the random forcing of stochastic parametrization schemes, that are typically motivated by subgrid‐scale variability, will be very useful (see, for example, *Düben and Dolaptchiev* [2015] for simulations with the 1‐D Burgers' equation).

Second, we show results for simulations for which precision is reduced with increasing forecast lead time that suggest that a reduction in precision shows a much smaller proportional effect on the forecast error if precision is only reduced later during the forecast, when model simulations already show an accumulated model error, compared to simulations for which precision is reduced already at the beginning of the forecast. A comparison between the change of the model simulation due to reduced precision and the model error compared to the true system can provide useful information on the minimal lead time at which precision can be reduced with only minor effects on the quality of model results. A reduction of precision with lead time appears to be very interesting for weather forecasts, especially since forecast models are sometimes reduced to lower resolution after a couple of days to save computing resources (see section 1 for an example for a reduction in the Ensemble Prediction System of the ECMWF after 10 days). Such a reduction in precision could be a good alternative for a reduction in resolution to save computing power and avoid imbalances and changes of boundary conditions (for example, in orography).

Third, we show that a speed‐up of a factor of 1.9 times is possible in comparison to the standard single precision FPGA configuration if numerical precision is reduced with hardly any increase in model error for short and long‐term simulations. If a small increase in model error can be accepted, precision can be reduced to a level that allows an increase in performance by a factor of 2.46. It is likely that an additional performance increase would be possible if the granularity of precision levels were increased by using more than two levels of precision in model simulations (such as **PX** and **PY**), to allow an even stronger reduction in precision in parts of the model. Results suggest that a simulation with reduced precision would allow an increase in resolution of atmosphere and ocean models. If large model configurations are considered ( $N_{x}=819,200$), single precision simulations with the CPU implementation are approximately a factor of 2 faster compared to double precision simulations and single precision simulations on the FPGA are a factor of 2.8 times faster compared to single precision simulations on two 6‐core CPUs. However, we do not claim that our CPU implementation achieved peak performance.

It has yet to be shown if large models of the size and complexity of realistic weather and climate models, with hundreds of thousands of lines of model code, can be ported to FPGA hardware. The success with the Lorenz model does not imply that the same approach can be taken for actual weather and climate models. Programming FPGAs is still much more complicated and consumes significantly more time than programming CPUs or even GPUs. Additionally, programming habits would need to change since the structure of code for FPGAs and CPUs is fairly different. However, the improvement in performance and the savings in power consumption due to the use of FPGAs appear to be very promising and the possibility of reducing precision to gain further performance is very attractive for geophysical modeling. Recent progress in the use of domain‐specific languages in the development of earth‐system models [e.g., *Torres et al*., 2013; *Fuhrer et al*., 2014] will make it easier to port large model setups to programmable hardware. We believe that larger model setups and dynamical cores of full global circulation models for atmosphere and ocean should be tested on FPGA hardware with reduced precision in the near future, following the work of *Oriato et al*. [2012], *Gan et al*. [2013], *Russell et al*. [2015], and this paper.

## Figures and Tables

**Figure 9:**
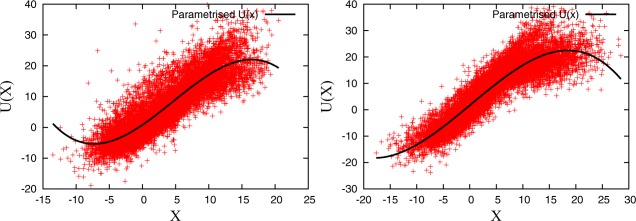
U(X) of the parametrized model for (left) the **C4** configuration and (right) the **C10** configuration and diagnosed data points for $\frac{hc}{b}\sum_{j=1}^{32}Y_{j,k}$ (red).

## Appendix A Parametrization of Small‐Scale Quantities

We introduce the two model configurations that have parametrized small‐scale quantities. Here the model is reduced to the large‐scale quantities. Interactions with small‐scale quantities are replaced by appropriate forcing terms that are added to the governing equation. To this end, we follow the same steps as *Wilks* [2005] and *Arnold et al*. [2013] and replace the interaction term with the small‐scale quantities in equation (1) by a polynomial:

$$\begin{array}{c} \frac{dX_{k}}{dt}=-X_{k-1}\left(X_{k-2}-X_{k+1}\right)-X_{k}+F-U(X_{k}),\\ U(X_{k})=a_{3}X_{k}^{3}+a_{2}X_{k}^{2}+a_{1}X_{k}+a_{0}, \end{array}$$

where $U(X_{k})$ tries to mimic the interactions to the missing *Y* variables if all information about the small‐scale quantities is lost. We find $U(X_{k})$ by fitting the polynomial to data points of the interaction term $\frac{hc}{b}\sum_{j=1}^{N_{y}}Y_{j,k}$ at different values of *X* that were diagnosed from 5000 time steps of a control simulation. Figure 9 shows a subset of the diagnosed data points and the fitted polynomials. We use $a_{3}=-0.0017,\;a_{2}=0.0013,\;a_{1}=1.6740$, and $a_{0}=1.6821$ for the **C4** configuration and $a_{3}=-0.0041,\;a_{2}=0.0560,\;a_{1}=1.4744$, and $a_{0}=0.8059$ for the **C10** configuration.
